# Lessons learnt during a complex, multicentre cluster randomised controlled trial: the ProAct65+ trial

**DOI:** 10.1186/1745-6215-14-192

**Published:** 2013-07-01

**Authors:** Zoe Stevens, Hannah Carpenter, Sheena Gawler, Carolyn Belcher, Deborah Haworth, Denise Kendrick, Richard Morris, Tahir Masud, Dawn A Skelton, Steve Iliffe

**Affiliations:** 1 Research Department of Primary Care and Population Health, UCL Medical School, Royal Free Hospital, London, NW3 2PF, UK; 2 School of Community Health Sciences, Division of Primary Care, Room 1309, Tower Building, University Park, University of Nottingham, Nottingham, NG7 2RD, UK; 3 Nottingham University Hospitals NHS Trust and University of Derby, Hucknall Road, Nottingham, NG5 1PB, UK; 4 School of Health & Life Sciences, Glasgow Caledonian University, A230 Govan Mbeki Building, Glasgow Caledonian University, Cowcaddens Road, Glasgow, G4 0BA, Scotland

**Keywords:** Randomised controlled trial, General practice, Recruitment, Adverse events, Exercise, Peer mentors, Postural stability instructors, Data collection, Quality assurance

## Abstract

**Background:**

Failure to recruit to target or schedule is common in randomized controlled trials (RCTs). Innovative interventions are not always fully developed before being tested, and maintenance of fidelity to the intervention during trials can be problematic. Missing data can compromise analyses, and inaccurate capture of risks to participants can influence reporting of intervention harms and benefits.

In this paper we describe how challenges of recruitment and retention of participants, standardisation and quality control of interventions and capture of adverse events were overcome in the ProAct65+ cluster RCT. This trial compared class-based and home-based exercise with usual care in people aged 65 years and over, recruited through general practice. The home-based exercise participants were supported by Peer Mentors.

**Results:**

(1) Organisational factors, including room availability in general practices, slowed participant recruitment so the recruitment period was extended and the number invited to participate increased. (2) Telephone pre-screening was introduced to exclude potential participants who were already very active and those who were frequent fallers. (3) Recruitment of volunteer peer mentors was difficult and time consuming and their acceptable case load less than expected. Lowering the age limit for peer mentors and reducing their contact schedule with participants did not improve recruitment. (4) Fidelity to the group intervention was optimised by introducing quality assurance observation of classes by experienced exercise instructors. (5) Diaries were used to capture data on falls, service use and other exercise-related costs, but completion was variable so their frequency was reduced. (6) Classification of adverse events differed between research sites so all events were assessed by both sites and discrepancies discussed.

**Conclusions:**

Recruitment rates for trials in general practice may be limited by organisational factors and longer recruitment periods should be allowed for. Exercise studies may be attractive to those who least need them; additional screening measures can be employed to avoid assessment of ineligible participants. Enrolment of peer mentors for intervention support is challenging and needs to be separately tested for feasibility. Standardisation of exercise interventions is problematic when exercise programmes are tailored to participants’ capabilities; quality assurance observations may assure fidelity of the intervention. Data collection by diaries can be burdensome to participants, resulting in variable and incomplete data capture; compromises in completion frequency may reduce missing data. Risk assessments are essential in exercise promotion studies, but categorisation of risks can vary between assessors; methods for their standardisation can be developed.

**Trial registration:**

ISRCTN43453770

## Update

### Background

The ProAct65+ trial is a three-arm parallel design cluster randomised controlled trial (RCT) of class-based exercises (Falls Management Exercise (FaME)), home-based exercises (Otago Exercise Programme (OEP)) facilitated by peer mentors, and usual care among people aged 65 years and over recruited from general practice [1]. The intervention ran for 24 weeks and in the initial research plan participants were to be followed up for 2 years from the end of the intervention. The trial’s primary outcome is the achievement of the current physical activity recommendations [2], at 1 year after the intervention ceased. Secondary outcomes include functional ability, falls and fear of falling, and quality of life. The trial is based in north and west London and Nottinghamshire/Derbyshire.

The unique characteristics of this trial are its recruitment base in general practice, its use of volunteer peer mentors to motivate participants in the OEP arm, its three-arm comparison in a UK setting, and its long follow-up period. This update paper describes the challenges faced during the ProAct65+ trial and modifications made to the trial protocol [1], during the trial.

The protocol was amended to recruit the target number of participants by extending the recruitment period and increasing the numbers of practices and patients per practice invited. To reduce the numbers excluded at baseline assessment, patient telephone screening prior to this initial assessment was introduced. Due to low recruitment and enrolment of peer mentors in the OEP arm the criteria for their recruitment and the intensity of their role were changed. A quality control system was incorporated into the FaME arm to aid standardisation of class activities. To reduce the burden of diary completion and optimise data collection on falls, service use and costs, the number of diaries during the follow-up period was reduced. To ensure governance of risks to participants an adverse event typology was developed and a system for checking it was applied consistently between sites.

The knowledge gained during the ProAct65+ trial and the modifications to its protocol may inform both future exercise trials and public health interventions with older people in general practice.

### Recruitment of general practices and participants

**Figure 1:**
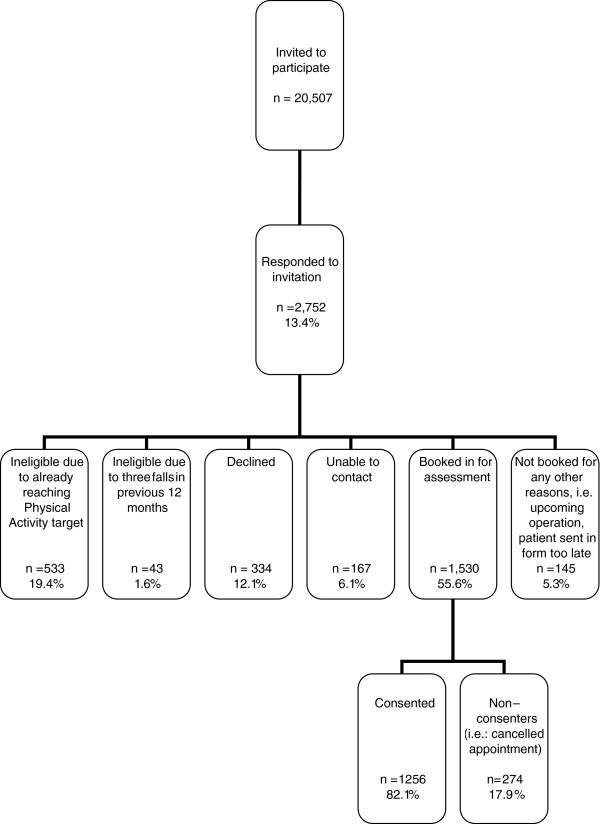
**Flow diagram of participant recruitment after alteration of recruitment processes.** Percentages refer to proportions in the next higher level of the recruitment process.

The flow path of participant recruitment to the trial is shown in Figure 1. The trial initially aimed to recruit 30 practices (15 at each site) and 40 patients per practice over a period of three weeks, to achieve a sample size of 1,200 participants aged 65 years and over. Based on the trial’s power calculation [1], at the outset 450 randomly selected eligible patients from each practice were invited by post by the practice, to express an interest in taking part. The proportion of those who expressed an interest varied between practices, from 8% to 19% in London and 7% to 21% in Nottinghamshire/Derbyshire, with a mean of 13.4%. Reminder postal invitations were considered to increase the expression of interest rate. Due to time constraints within the general practices however, reminder invitations were only posted from three practices in London and 11 practices in Nottinghamshire/Derbyshire. An additional recruitment method of displaying posters in general practices was considered, however it was not implemented to avoid complicating selection biases among participants. In order to achieve the recruitment target, the number of invitations to eligible patients was increased to 600 from each practice to adjust for the lower than anticipated recruitment rate in practices where 450 were approached. When this change was implemented no change in response was seen (range: 5% to 22% in London, 7% to 26% in Nottinghamshire/Derbyshire), but the number recruited increased due a larger study population being available.

Stratified random sampling was planned, whereby eligible patients would be stratified into age groups 65 to 74 years and 75+ years. To simplify the tasks for the practices and to encourage their cooperation this stratified sampling approach was abandoned and patients were sampled from one list of patients aged 65 years and over.

**Figure 2:**
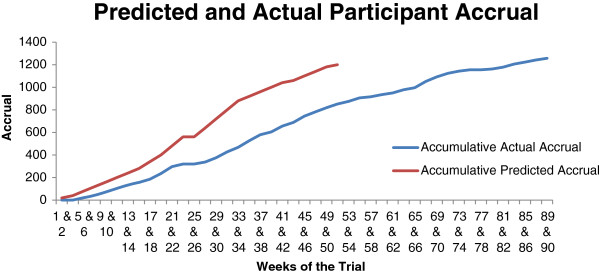
**Recruitment of participants to the trial over time.** The graph shows participant recruitment was slower than anticipated and numbers recruited were lower in the timeframe originally allocated to recruitment. The recruitment period should have ended in week 52, however it was extended to allow more practices to be recruited to reach the participant recruitment target. In week 13 the number of participants invited from each practice increased from 450 to 600 and the telephone eligibility screen was implemented.

Despite the increase in invitations to 600 patients per practice, the number recruited from our first 30 general practices was below the target level (based on the sample size calculation and the timetable for recruitment) at both sites (469 in London and 417 in Nottinghamshire/Derbyshire). In part, this was due to patients who expressed interest in the trial being frequent fallers (three or more falls in the previous 12 months), either already reaching the physical activity target or meeting other exclusion criteria, listed in the trial protocol [1]. Additionally, room availability in practices for baseline assessments was limited and it took up to 6 weeks in some practices to assess and recruit the target number of participants. The recruitment phase of the trial was 9 months longer than anticipated because of the need to recruit more practices at both sites and to allow more time at each practice to undertake recruitment. In total 43 general practices and 1,256 participants were finally recruited (see Figure 2): 22 practices and 605 participants in London and 21 practices and 651 participants in Nottinghamshire/Derbyshire. The extension of the recruitment period altered the timescale of the trial and potentially limited data collection for the 18- and 24-month follow-up.

### Adding an eligibility screen

Although there were multiple screening steps for eligible patients (by electronic and manual patient searches by the general practices), initially researchers met some patients at the baseline assessment/consent stage who were ineligible due to meeting exclusion criteria, principally falling more than three times in the previous 12 months or already exercising at the target level of five sessions of 30 min of moderate exercise per week. To limit assessment of the ineligible, when arranging the baseline assessment appointment, researchers asked questions over the telephone about falls in the last year and current levels of exercise. Only if the patient was still potentially eligible were they invited to the baseline assessment.

### Implementing the interventions

#### Otago home-based exercise programme (OEP)

Participants were taught the exercise programme in a single group session at a community venue by an exercise instructor trained in the OEP and given a tailored manual of exercises to do at home. In London, 31 sessions were taught to a mean of seven participants per session over 15 months. In Nottinghamshire/Derbyshire, 18 sessions were taught to a mean of seven participants per session over 10 months. Participants were given ankle weights starting at 1 kg and progressing up to 4 kg. Some participants were unable to attend the session due to other personal commitments and were visited and trained by the exercise specialist in their home.

**Figure 3:**
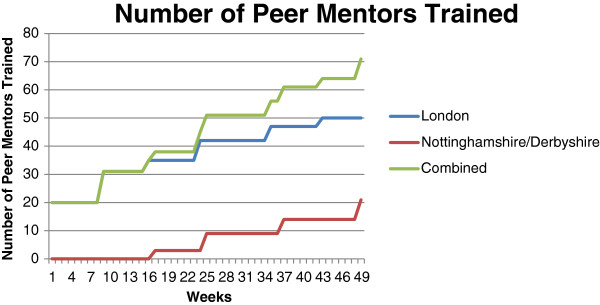
**Number of trained peer mentors over time.** This graph shows the number of fully trained peer mentors at each site, and combined, over time. It took 43 weeks in London to achieve 50 trained peer mentors, and 49 weeks in Nottinghamshire/Derbyshire to train 21 trained peer mentors.

Volunteer peer mentors were recruited to support the participants during the exercise programme (Stevens Z, Barlow C, Iliffe S: Promoting physical activity amongst older people in primary care using peer mentors, Submitted). The aim was to recruit 50 peer mentors at each site, with the expectation that each would mentor four to five participants. Initially peer mentor recruitment was aimed at adults aged 65 years and over. Peer mentors were recruited by displaying posters and flyers in libraries, approaching senior exercise classes, through articles in local newspapers and stories on local radio, by word of mouth from trained volunteers and by invitations in participating general practices. Recruitment was slow (see Figure 3) and time consuming. Despite intense efforts the number of peer mentors who joined the trial did not reach the target. After eight months of peer mentor recruitment the age criterion for peer mentors was altered to allow the enrolment of adults aged 50 and over. This led to an additional eight peer mentors being enrolled in London but no more in Nottinghamshire/Derbyshire.

Table 1 shows the length of time spent on recruiting peer mentors, numbers of individuals who expressed an interest in becoming a peer mentor, numbers of individuals trained, the number who subsequently disengaged from the study, and the final number of peer mentors who volunteered and were allocated participants. There is a large difference in the number of people who expressed an interest in becoming a peer mentor and those that were trained; individuals decided whether or not they wanted to join a training session after receiving an information sheet about what the volunteering entailed and learning where they would need to travel to when visiting participants. Feedback from peer mentors suggests that disengagement was due in part to the length of time between training and beginning work. This was due to the time needed to obtain research management and governance approvals for the peer mentors, and for the randomisation of the practices and the start of interventions. Disengagement was also due in part to the distance peer mentors would need to travel to support participants.

**Table 1:** Peer mentors recruitment, training and deployment

	**London**	**Nottinghamshire/Derbyshire**
Time spent on recruitment by staff (months)	12	15
Expressed interest (*n*)	130	79
Trained (*n*)	50	21
Disengaged (*n*)	19	14
Volunteered (*n*)	31	7
Time from trained to deployed (days)	Mean 132 (range, 21-255)	Mean 155 (range, 75-257)

This table shows the time taken to recruit peer mentors in both trial sites, the numbers of individuals who expressed an interest in becoming a peer mentor, those who were trained, disengaged and volunteered, and the time it took from when peer mentors were trained to when they began their volunteering at both trial sites.

Each peer mentor in the trial mentored a mean of three participants (range, 1-13) in London, and a mean of three participants (range, 1-5) in Nottinghamshire/Derbyshire. Overall, both sites therefore fell short of the target of four to five participants per peer mentor. All participants, regardless of their peer mentor support, received the initial exercise training session and a booklet with tailored exercise instructions. Not all participants received a peer mentor because of the difficulties recruiting them. In London 123 (53%) participants and in Nottinghamshire/Derbyshire 21 (12%) participants had a peer mentor. Despite using the same recruitment methods, recruitment difficulties were greater in Nottinghamshire/Derbyshire. This may have been because the trial was competing with existing peer mentor physical activity programmes for older people in Nottinghamshire/Derbyshire. Due to the difficulty recruiting sufficient peer mentors for each participant, we debated using either Postural Stability Instructors (PSIs), already working on and familiar with the study, or volunteers aged < 50 years, who would be trained in the delivery of the home based programme. The Trial Steering Committee advised to keep the intervention true to usual practice in the NHS, that is, one instruction session plus a manual of exercises. Therefore, where there were insufficient peer mentors for all participants, they were not supplemented by an alternative person and some participants had no peer mentor support at all.

In another attempt to increase the number of peer mentors and encourage them to support more participants, the number of their supportive contacts with participants was reduced. Initially peer mentors were scheduled to visit participants in their home on four occasions and telephone them 12 times during the 24-week intervention. This was reduced to two visits and eight telephone calls. Over both sites, the number of home visits ranged from zero to five (mean two), and the number of telephone call contacts ranged from zero to 18 (mean six). Modification of the number of contacts did not increase peer mentor recruitment or their case load.

#### Falls management exercise programme (FaME)

The FaME intervention is a weekly group-based exercise session, supplemented with additional home exercises (modified from the OEP) described in a booklet. PSIs were recruited to lead the classes. The trial aimed to recruit 12 PSIs per site. In London 16 PSIs were recruited with a total of seven working on the trial. As there were few qualified PSIs available to recruit in Nottinghamshire/Derbyshire, the trial recruited and trained physiotherapists and exercise professionals who were interested in becoming a PSI and working on the trial. Sixteen individuals embarked on the PSI training course (15 completed the training), and seven of them worked on the trial. Some PSIs were not employed on the trial due to their limited availability. Additionally the complex and lengthy process of completing research governance approvals resulted in losing some available PSIs. The recruitment target was reached with 32 PSIs recruited and trained over both sites. Of these 14 (44%) delivered the intervention, enabling the intervention to be fully staffed.

**Figure 4:**
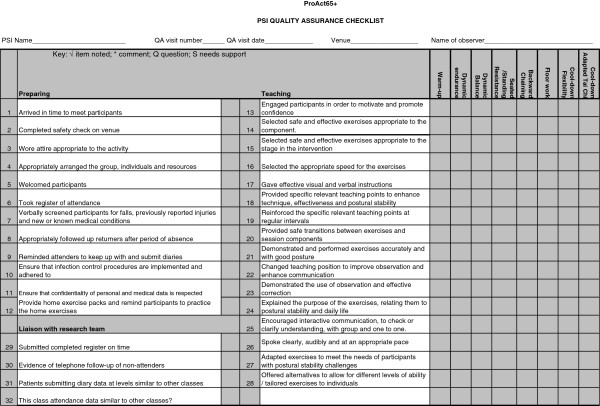
**Quality assurance checklist for FaME intervention.** This figure shows the checklist which was used to quality assure the FaME exercise classes and instructors.

All recruited PSIs received a trial specific ‘top-up’ training session, delivered by a dedicated staff member of the trial. This session aimed to prepare the PSIs, with various professional backgrounds, to deliver a standardized intervention to the trial participants. Additionally, in order to quality assure and standardise the FaME intervention, two quality assurance members of the trial oversaw the intervention delivery by attending four exercise sessions over the 24-week intervention period for each PSI in all of the FaME practices. The quality assurers went to the sessions individually, except the first two sessions when they attended together to standardise their method. Overall, 45 FaME classes in London and 38 in Nottinghamshire/Derbyshire were quality assured. Using a standard checklist (see Figure 4), the quality assurers observed the exercise class and then gave feedback and an action plan to the PSI leading the class to improve intervention delivery, optimize participants’ ability to undertake increasingly demanding exercises, and standardise the exercise intervention as much as possible.

### Measuring falls, service use and exercise

#### Logistics

Participants were seen and assessed by a researcher at baseline and at the end of the intervention period. During the intervention self-completion diaries were posted to participants every month. During the follow-up period participants were posted self-completion diaries every 3 months, larger self-completion questionnaires every 6 months, and telephoned for a short questionnaire every 6 months. See Table 2 for an outline of the various questionnaires at different time points. Full details of questionnaires are given in the trial protocol [1].

**Table 2:** Questionnaires used during the trial

	**Baseline**	**End of intervention**	**6-, 12-, 18-, 24-month follow-up**	**Diaries x14**
Face to face	Y	Y		
Telephone	Y	Y	Y	
Postal	Y	Y	Y	Y

This table shows how data was collected from participants and how often.

Because non-monetary incentives are known to assist retention in trials [3], small incentives were sent to participants to encourage completion of postal questionnaires. With diary six and 12, participants received a ProAct65+ pen, and with diary eight and 10, they received a ProAct65+ cotton shopping bag. Participants were also sent an annual Christmas card and brief newsletters with each diary they received.

Research staff at both sites telephoned participants every three months to remind them to return questionnaires. Up to three contacts with participants were made to undertake each telephone interview. Some participants did not return self-completion diaries and/or questionnaires and some were not available for a telephone interview due to a variety of reasons, including being on holiday, at work, too busy, or forgetting or losing the questionnaires (Table 3).

**Table 3:** Response rates at 12-month follow-up

	**Postal**	**Telephone**
London	66%	79%
Nottinghamshire/Derbyshire	76%	82%
Both sites	72%	80%

This table shows the response rates to the postal and telephone questionnaires, from participants remaining in the trial at 12-month follow-up.

The self-completion diaries requested information on participants’ health and social service use, falls and current exercise levels [4]. Initially it was planned for participants to receive monthly prospective diaries to complete throughout the full length of the trial. When participants said that they wished to withdraw from the trial due to the quantity and frequency of questionnaires they were offered the opportunity to remain in the trial but complete only the six-monthly questionnaires, and not receive further diaries. By doing this the trial retained 52 participants in London, and 28 in Nottinghamshire/Derbyshire (6% of total trial participants) who would otherwise have withdrawn from the trial. To further limit the number of participants who withdrew from the trial due to the burden of the questionnaires and diaries the frequency of the diaries sent during the 2-year follow-up phase was reduced from monthly to quarterly. The diaries sent during the follow-up phase required the participants to recall their service use and falls from the last 3 months and record a 1-week prospective ‘snap-shot’ of their exercise activities.

#### Capturing adverse events

**Figure 5:**
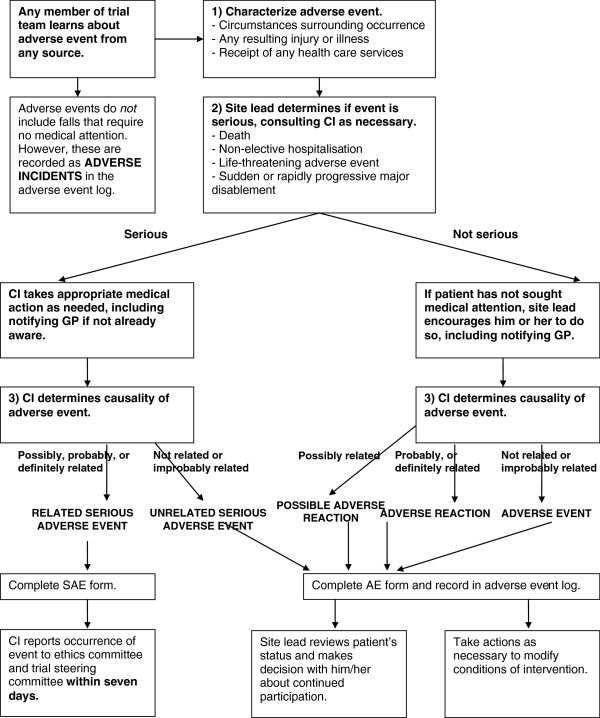
ProAct65+ risk management pathway.

Adverse events were monitored throughout the trial to assess the trial’s safety and manage participant risks. This is especially important since exercise within this age group may be associated with an increased risk of falls [5,6]. The ProAct65+ trial used a risk management pathway for capturing, classifying and dealing with participant adverse events (Figure 5), which initially categorised all occurrences as Serious Adverse Events (SAEs), Adverse Events (AEs), Adverse Reactions (ARs) or Adverse Incidents. All data were logged and any SAEs were reported to the Trial Steering Committee. The original risk management pathway and the definitions of events, reactions and incidents are reported in the trial protocol [1].

A comparison of all events between trial sites was carried out towards the end of the trial’s intervention phase. There were noticeable differences in the numbers of Adverse Reactions recorded between sites with London categorising 5%, and Nottinghamshire/Derbyshire categorising 16% of their total events as Adverse Reactions.

A cross-checking system was therefore implemented between sites in an attempt to standardise categorisation. All events from each site, except ‘Adverse Incidents’ , were checked by the other site. If the other site’s categorisation was different to the original categorisation, this was deemed a ‘mismatch’. Mismatches between sites were identified, and blinded forms then passed to the Principal Investigators who discussed and agreed a final categorisation. The initial calculation of mismatches was performed towards the end of the intervention phase, when there were 51 mismatches, giving a mismatch rate between sites of 19%.

The decision on whether an event is ‘Possibly Related’ to the trial is open to subjective interpretation. Consequently, 45 of the 51 (88%) discrepancies in the categorisation of events recorded at each site were between Adverse Events and Adverse Reactions. The category ‘Possible Adverse Reaction’ (Possible AR) was therefore added. After the introduction of the Possible Adverse Reaction category, the mismatch rate (prior to discussion between Principal Investigators) fell to 2.6%.

After advice from the Trial’s Steering Committee, the categorisation was further modified to enable Serious Unrelated Adverse Events to be distinguished from Non-Serious Adverse Events. The final categories applied to the trial’s events were therefore: Serious Adverse Events; Unrelated Serious Adverse Events; Adverse Events; Adverse Reactions; Possible Adverse Reactions; Adverse Incidents.

### Discussion and implications

The challenges faced during the ProAct65+ trial and solutions to these challenges are summarised below. Other research which has faced similar challenges is discussed here for comparison. Implications for future research and public health work are suggested.

Although the trial over-achieved its recruitment target, the recruitment process was more difficult and slower than anticipated. The time needed to recruit participants was underestimated and an extension in recruitment time was vital. Other trials recruiting from general practice have found similar slow and difficult recruitment, with lower than anticipated numbers recruited and required time extensions [7-9]. In ProAct65+, the recruitment phase was extended, more general practices were recruited, and more patients at each practice were invited to participate. Trials in general practice should expect the recruitment phase to be longer than initially anticipated, and for more general practices and participants to be invited, to achieve the target numbers. It is advisable to keep recruitment as straight forward as possible and to minimize the work needed by general practices [9,10].

Expressions of interest were received from patients already exercising at the target level of 150 min of moderate activity per week, and from frequent fallers. It is likely that most exercise trials receive interest from a proportion of the population which is atypical, (that is, more active), than the general population [11]. Telephone pre-screening was introduced to exclude such ineligible patients before they reached the baseline assessment appointment.

The use of volunteers to act as peer mentors proved complicated. As found with other interventions using volunteers, recruitment was slow and the numbers deployed were low [12]. Additionally peer mentors had accepted a lower than expected case load, and consequently support for participants was diluted [13-15], which threatens the OEP intervention’s effectiveness. The lower age limit for peer mentors was reduced, as was the frequency of their contacts with participants, but with only limited benefit. It is important for future interventions to allow enough time and resources (human and financial) when planning peer mentor programmes. In order to minimize the time from training a peer mentor to deployment and to retain interested volunteers, attention needs to be focused on speeding up the process of gaining Criminal Record Bureau checks and Research Management and Governance approvals. Strategies to optimise peer mentor motivation and involvement need further investigation. Additionally, the number of supportive contacts between peer mentors and participants varied and often differed from the number of contacts advised by the research team, which may reflect the needs of the individual participants. Future projects implementing peer mentor support should be aware of participants needs for more or less support, which may lead to varied numbers of contacts with peer mentors.

Failure to ensure the fidelity of interventions is an important source of variation affecting the credibility and utility of research [16]. Trial specific ‘top-up’ training was delivered to instructors in advance of the interventions. Quality assurance observation visits to classes were carried out by expert instructors, with verbal and written feedback on performance. Exercise instructors may not always achieve a balance between tailoring exercise and providing a standardised programme, and observations of intervention delivery and receipt are recommended [17].

Participants can be burdened by frequent data collection which causes variable response rates to the self-completion questionnaires [4]. Compromises in the frequency of data collection were made, and the frequency of the self-completion diaries was reduced. It has however been suggested that maintaining between-assessment contacts is important to reduce attrition [18]. Personal contact with the research team is said to improve response rates [19], as do reminders, incentives and printed educational materials [20,21]. Although more time-consuming and costly than postal self-completion questionnaires, it has previously been shown that home visits to collect follow-up data are useful and can reduce attrition bias in longitudinal studies [22]. Alternatively, higher response rates to postal questionnaires have been found when they are sent directly by the general practices rather than by the research team; this may also be a method to aid retention of participants during a trial [23].

The classification of safety events between sites was variable, so a method of cross-checking and standardisation was developed. Both site Principal Investigators reviewed and discussed discrepancies in categorisation and a new Possible Adverse Reaction category was introduced to reduce variability. This method of cross checking and the classifications of safety events used in ProAct65+ could be applied to future exercise or indeed any multisite trials.

The ProAct65+ trial is a large pragmatic randomised controlled trial. Despite difficulties the trial reached its recruitment target, making it the largest exercise trial to date in British general practice. The trial’s flexibility in being able to adapt to unexpected problems may have led to the trial’s success [8]. The lessons learnt during the ProAct65+ trial have been valuable and have potential implications for similar trials in general practice.

## Competing interests

All authors declare they have no competing interests.

## Authors’ contributions

ZS made substantial contributions to the acquisition of data within the manuscript. ZS drafted, critically revised and finalised the manuscript. HC, SG and CB made substantial contributions to the acquisition of data within the manuscript and made revisions to the manuscript. DH contributed to the acquisition of data and critically revised the manuscript for important intellectual content. DK, RM, TM and DAS made substantial contributions to the design of the trial and critically revised drafts of the manuscript for important intellectual content. SI developed the concept and design of the trial and manuscript. SI drafted and critically revised the manuscript for important intellectual content. All authors read and approved the final manuscript.
